# Influenza virus surveillance in Argentina during the 2012 season: antigenic characterization, genetic analysis and antiviral susceptibility

**DOI:** 10.1017/S0950268815001806

**Published:** 2015-09-08

**Authors:** E. BENEDETTI, R. S. DANIELS, A. PONTORIERO, M. RUSSO, M. AVARO, A. CZECH, A. CAMPOS, N. PERIOLO, V. GREGORY, J. W. McCAULEY, E. G. BAUMEISTER

**Affiliations:** 1 National Influenza Centre PAHO/WHO, Servicio Virosis Respiratorias, Departamento Virología, Instituto Nacional de Enfermedades Infecciosas, ANLIS ‘Carlos G. Malbrán’, Buenos Aires, Argentina; 2 The Francis Crick Institute, Mill Hill Laboratory, The Ridgeway, Mill Hill, London, UK

**Keywords:** Influenza, influenza vaccines, resistance to drugs, respiratory infections, surveillance

## Abstract

The activity and circulation of influenza viruses in Argentina was studied during 2012 as part of the Argentinean Surveillance for Influenza and other Respiratory Viruses, in the context of Global Influenza Surveillance. The antigenicity and molecular characteristics of haemagglutinins (HA) of circulating influenza A and B viruses were analysed to assess the emergence of virus variants. Susceptibility to oseltamivir and zanamivir was evaluated by enzymatic assay and results were backed-up by sequencing of the neuraminidase (NA) genes. During the 2012 season, influenza virus circulation in Argentina was detected from weeks 24 to 51. The HA sequences of the studied A(H1N1)pdm09 subtype viruses segregated in a different genetic group compared to those identified during the 2009 pandemic, although they were still closely related antigenically to the vaccine virus A/California/07/2009. The HA sequences of the A(H3N2) viruses analysed fell into the A/Victoria/208/2009 clade, genetic group 3C. A mixed circulation of virus variants belonging to B/Victoria and B/Yamagata lineages was detected, with B/Victoria being dominant. All viruses tested were sensitive to oseltamivir and zanamivir except one. This isolate, an A(H1N1)pdm09 virus possessing the substitution NA-N295S, showed highly reduced inhibition by oseltamivir and reduced inhibition by zanamivir. Virological and epidemiological surveillance remains critical for detection of evolving influenza viruses.

## INTRODUCTION

Seasonal influenza is a public health problem that affects 5–20% of the world's population annually causing high morbidity and mortality, especially in risk groups [1], and the range of type A viruses, largely maintained in avian species, poses potential pandemic threats [2].

Types A and B influenza viruses cause epidemic disease in humans. Influenza A viruses are further classified into subtypes according to the characteristics of two surface glycoproteins: haemagglutinin (HA) and neuraminidase (NA). Currently, two lineages of influenza B viruses co-circulate. Influenza viruses display high rates of mutation, most marked in the genes encoding the glycoproteins HA and NA which are under the greatest selection pressures by host immune systems [3].

Minor changes in HA and NA can result in the emergence of antigenically drifted viruses. Antigenic drift of influenza A HA is a well-studied evolutionary process, in terms of both the timing and the amino acid locations of changes that lead to important antigenic variation [4]. Through zoonotic events, influenza A viruses can undergo antigenic shifts, antigenic changes of greater magnitude that are responsible for the emergence of new subtypes, which may result in a widespread pandemic in an immunologically naive human population [5]. Identification and characterization of circulating influenza viruses is essential for detection of the emergence of such antigenically drifted/shifted viruses in different regions of the world.

Trivalent inactivated influenza virus vaccines are widely used throughout the world, with approximately 300 million doses [6]. The antigen content of vaccines needs to be monitored on an annual basis and updated frequently, due to the variability of influenza viruses [7, 8]. The WHO coordinates influenza surveillance throughout the world, which is important for the detection and identification of newly emerging epidemic variants, and contributes to the biannual selection of appropriate vaccine strains [9]. The trivalent vaccine formulation recommended by WHO for the Southern Hemisphere 2012 influenza season contained the following: A/Perth/16/2009-like (H3N2), A/California/07/2009-like (H1N1)pdm09 and B/Brisbane/60/2008-like (Victoria lineage) virus components [10].

Vaccines are the most effective primary strategy available for preventing and lowering the impact of an influenza outbreak. Otherwise, antiviral drugs can be used in prevention and treatment of influenza, resulting in lower mortality and morbidity. Antiviral drugs for influenza currently include two classes: M2 ion channel inhibitors amantadine and rimantadine, and neuraminidase inhibitors (NAIs) oseltamivir (Tamiflu) and zanamivir (Relenza) [11, 12]. Prior to 2009, NAI use was largely restricted to Japan and the USA; the onset of the pandemic in 2009 brought with it a sharp increase in NAI usage in many countries [13]. With increased drug use, the likelihood of resistance developing increases. Due to the global spread of adamantane resistance, it has been recommended discontinue the use of M2 ion channel inhibitors. This showed that antiviral-resistant influenza viruses represent a genuine public health risk [13]. In the USA, from 2009 to 2011, 0·9–1·1% of influenza A(H1N1)pdm09 viruses and 0·2% of influenza A(H3N2) were oseltamivir resistant, while almost 100% of A(H1N1)pdm09 and A(H3N2) viruses were adamantane resistant. Therefore, surveillance for resistance is important in terms of prevention and treatment of influenza infection [11].

The aims of this study were to evaluate the 2012 influenza season in Argentina and determine the virus characteristics of a subset of circulating influenza A and B viruses to include monitoring of their susceptibility to antiviral drugs.

## MATERIALS AND METHODS

### Specimen collection and influenza detection/diagnosis

Weekly influenza virological surveillance in Argentina is conducted routinely by the WHO National Influenza Centre (NIC) located in Buenos Aires as part of the WHO's Global Influenza Surveillance and Response System (GISRS).

The NIC, as the National Laboratory for Influenza and Respiratory Viruses (NLIVR), conducts the surveillance activities with the National Laboratory Network, composed of 65 laboratories distributed throughout the country. This ensures efficient monitoring of virus circulation with approximately 70 000 respiratory samples per year being submitted for diagnostic testing. Nasopharyngeal aspirates and throat or nasal swabs from paediatric and adult outpatients and inpatients with acute respiratory infection are collected and examined by immunofluorescence assay (IFA) [14] for diagnosis of influenza A, influenza B, and other respiratory viruses, including respiratory syncytial virus (RSV), adenovirus, and parainfluenza virus. IFA negative samples are also studied by one-step real-time reverse transcriptase–polymerase chain reaction (rtRT–PCR) assay using primer/probe sets against regions of two different genes: the M gene of influenza type A virus and the nucleoprotein (NP) gene of influenza type B virus, using the Centers for Disease Control and Prevention (CDC, USA) influenza diagnosis protocols. The influenza-positive clinical samples, types A and B, are routinely submitted to the NIC for further characterization. In 2012, the NIC received almost 2400 influenza-positive samples from the national network.

### Subtype determination and virus isolation

All influenza A samples are routinely subtyped by one-step rtRT–PCR assay using specific primer/probe sets for the HA gene recommended by the CDC. Of the influenza-positive specimens received by the NIC in the 2012 influenza season, 824 (35%) were selected for isolation in mammalian cells taking into account the specimens’ collection dates, preservation conditions, and the geographical locations in order to obtain virus isolates from different regions of the country throughout the study period. Parental Madin–Darby canine kidney (MDCK) cells were used for isolation of A(H1N1)pdm09 and B viruses and MDCK-SIAT1 cells (engineered to express increased levels of sialyl-*α*_2,6_-galactose moieties) for A(H3N2) viruses [15]. MDCK and MDCK-SIAT1 cells were maintained in Minimum Essential Medium with Earle's salts, non-essential amino acids and l-glutamine (Gibco no. 41-500-034) and Dulbecco's Modified Eagle's Medium (Gibco no. 12 800-01) respectively, both supplemented with 2% fetal bovine serum (Gibco, USA). The presence of virus in tissue-culture supernatant was assessed by direct IFA and/or haemagglutination assay (HA) according to standard methods using suspensions of guinea pig red blood cells (GPRBC, 0·75% v/v) [16] performed no later than 7 days post-infection. Thirty-nine influenza isolates [two influenza A(H3N2), 26 A(H1N1)pdm09 and 11 B viruses] with collection dates in July and August [epidemiological weeks (EW) 27–35] from different provinces of the country with the purpose of achieving a geographical representation were sent to the WHO Collaborating Center for Reference and Research on Influenza (WHO CC), London, UK, for comprehensive antigenic, genetic and antiviral drug susceptibility studies.

Written informed consent and explicit ethical approval were not sought as this study was an observational undertaking as part of routine virological surveillance (performed anonymously, without identification of patients), as established in the terms of reference for WHO National Influenza Centres.

### Antigenic characterization

A total of 33 isolates were antigenically characterized by haemagglutination inhibition (HI) tests as follows: 20 influenza A(H1N1)pdm09, two A(H3N2) and 11 influenza B. HI testing was performed using a panel of post-infection ferret antisera using turkey red blood cells for H1N1 and B viruses [17]. For H3N2 viruses assays were performed with GPRBC in the presence of 20 nm oseltamivir to circumvent the NA-mediated binding of H3N2 viruses to the red blood cells [18].

### NAI susceptibility assay

Analysis of NA susceptibility was performed on 16 influenza A(H1N1)pdm09, two A(H3N2) and 10 influenza B isolates. NA activity was measured using the fluorescent substrate, 2’-(4-methylumbelliferyl)-*α*-d-*N*-acetylneuraminic acid (MUNANA; Sigma, USA) [19]. Briefly, 15 *μ*l virus was incubated with 30 *μ*l of 100 *μ*m MUNANA in 32·5 mm MES buffer (pH 6·5) containing 4 mm CaCl_2_ for 1 h at 37 °C. The reaction was stopped by addition of 150 *μ*l of 0·14 m NaOH in 83% ethanol and fluorescence of the released 4-methylumbelliferone was measured at excitation and emission wavelengths of 365 nm and 450 nm, respectively. The activity of each virus sample was titrated, by assaying serial twofold dilutions and virus suspensions were adjusted to equivalent NA activities, which fell in the linear portion of the activity curve. Each virus was pre-incubated for 30 min at 37 °C with oseltamivir or zanamivir at final concentrations of 5 *μ*m-0·05 pm, in serial tenfold dilutions, NA activity measured and the drug concentration that inhibited 50% of the neuraminidase (IC_50_) was determined [20].

### Gene sequencing and analysis

RNA was extracted from propagated viruses and HA and NA genes amplified using one-step rtRT–PCR. Gene sequences were determined by the Sanger method using an ABI 3730XL capillary sequencer. Sequences of primers used for gene amplification and Sanger sequencing can be obtained on request. Phylogenetic analysis of the nucleotide sequences was performed using RaxML (sco.h-its.org/exelixis/web/software/raxml/) and the dendograms generated using FigTree (http://tree.bio.ed.ac.uk/software/figtree/). All sequences determined in the course of this work have been deposited in the Global Initiative on Sharing All Influenza Data (GISAID) database under the following virus identifiers: EPI_ISL_131 955, EPI_ISL_131 956, EPI_ISL_131 957, EPI_ISL_131 958, EPI_ISL_131 960, EPI_ISL_131 964, EPI_ISL_131 965, EPI_ISL_131 966, EPI_ISL_131 969, EPI_ISL_131 970, EPI_ISL_131 973, EPI_ISL_131 974, EPI_ISL_131 975, EPI_ISL_131 976, EPI_ISL_131 977, EPI_ISL_131 978, EPI_ISL_131 979, EPI_ISL_131 980, EPI_ISL_131 981, EPI_ISL_31 982, EPI_ISL_131 983, EPI_ISL_131 984, EPI_ISL_131 985, EPI_ISL_131 991, EPI_ISL_131 992, EPI_ISL_132 002, EPI_ISL_132 003, EPI_ISL_132 251, EPI_ISL_132 252, EPI_ISL_132 253, EPI_ISL_132 257, EPI_ISL_132 273, EPI_ISL_132 293, EPI_ISL_132 294, EPI_ISL_132 295, EPI_ISL_132 296, EPI_ISL_132 297.

## RESULTS

### Virological surveillance

**Fig. 1.**
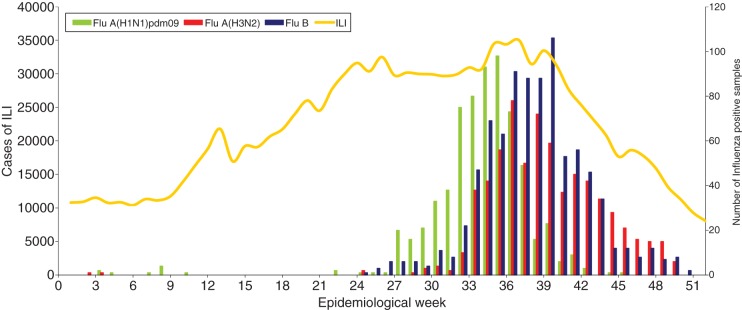
Influenza-like illness (ILI) surveillance and influenza-positive samples submitted to the National Influenza Centre, Buenos Aires throughout 2012 in Argentina.

For 2012, according to information provided by the Argentinean Ministry of Health (MoH), a total of 68 225 cases of acute respiratory infection were studied in the laboratory according to the National database (SIVILA), 22 745 were positive for respiratory viruses. RSV was the most common virus detected, accounting for 74·9% of the positive cases, followed by influenza viruses (12·6%) and parainfluenza virus (6·5%) [21]. The NIC received 2387/2859 (83·5%) of the influenza-positive samples detected in the country, of which 2212 were typed/subtyped. Influenza A(H3N2), A(H1N1)pdm09 and B viruses were detected from January to December in similar proportions. In 2012, as in previous seasons, influenza activity peaked late (middle August to late September). Influenza A(H1N1)pdm09 viruses (666, 30·1%) were detected sporadically from EW 1–26, increasing from EW 27–40 with peak detection in EW 36 (early September), while influenza A(H3N2) viruses (677, 30·6%) were detected from June to December with peak detection in EW 37 (mid-September). Influenza B viruses (869, 39·3%) were detected from EW 25, with peak detection in EW 40 (early October) (Fig. 1).

### Antigenic and genetic characteristics of the viruses

In 2012, the average rate of influenza isolation in tissue culture at the NIC was 56%. A subset of the isolates from the early part of the season, EW 27–35 (Fig. 1), were antigenically and genetically characterized at the WHO CC, London, UK. The results of HI assays performed on influenza A and B isolates are presented in Tables 1–4.

**Table 1.** Antigenic analyses of A(H1N1)pdm09 viruses

				Haemagglutination inhibition titre							
				Post-infection ferret antisera							
		Collection date	Passage history	A/California/7/09 F29/11	A/Bayern/69/09 F11/11	A/Lviv/N9/09 C4/34/09	A/Christchurch/16/10 F30/10	A/Hong Kong/3934/11 F21/11	A/Astrakhan/1/11 F22/11	A/St. Petersburg/27/11 F23/11	A/St. Petersburg/27/11 F24/11

	Genetic group …						4	3	6	7	6
**Reference viruses**											
A/California/7/2009		2009-04-09	E1/E2	*1280*	1280	1280	640	640	640	640	1280
A/Bayern/69/2009		2009-07-01	MDCK4/MDCK1	80	*320*	160	80	40	80	80	80
A/Lviv/N6/2009		2009-10-27	MDCK4/SIAT1/MDCK2	640	1280	*1280*	320	160	320	320	160
A/Christchurch/16/2010	4	2010-07-12	E2/E2	1280	1280	1280	*5120*	1280	1280	1280	2560
A/Hong Kong/3934/2011	3	2011-03-29	MDCK2/MDCK2	640	160	320	640	*1280*	1280	640	1280
A/Astrakhan/1/2011	6	2011-02-28	MDCK1/MDCK5	1280	640	1280	1280	2560	*2560*	1280	5120
A/St. Petersburg/27/2011	7	2011-02-14	E1/E2	5120	1280	5120	2560	2560	5120	*5120*	5120
A/St. Petersburg/100/2011	6	2011-03-14	E1/E2	2560	1280	2560	1280	2560	2560	2560	*5120*
**Test viruses**											
A/Salta/1122/2012	6	2012-07-14	MDCK2/MDCK1	640	640	640	320	320	640	320	640
A/Salta/1121/2012	6	2012-07-15	MDCK2/MDCK1	1280	640	1280	12580	2560	2560	2560	5120
A/Santa Fe/333 171/2012	6	2012-07-20	MDCK1/MDCK1	2560	1280	1280	2560	2560	2560	2560	5120
A/Salta/1363/2012	6	2012-07-26	MDCK2/MDCK1	2560	1280	1280	2560	2560	2560	2560	5120
A/Salta/1341/2012	6	2012-07-26	MDCK2/MDCK1	2560	1280	1280	2560	2560	2560	2560	5120
A/Tucumán/59 232/2012	6	2012-07-27	MDCK2/MDCK1	1280	640	1280	1280	2560	2560	2560	5120
A/Corrientes/66 335/2012	6	2012-08-03	SIAT2/MDCK1	2560	1280	2560	2560	2560	2560	5120	5120
A/Formosa/V2361/2012	6	2012-08-03	SIAT2/MDCK2	5120	2560	5120	5120	5120	5120	5120	5120
A/Tucumán/I1956/2012	6	2012-08-07	MDCK2/MDCK1	2560	640	1280	2560	2560	2560	2560	5120
A/Misiones/6563/2012	6	2012-08-07	SIAT2/MDCK1	1280	640	1280	2560	2560	2560	2560	5120
A/Misiones/6592/2012	6	2012-08-08	MDCK2/MDCK1	1280	640	1280	1280	2560	2560	2560	5120
A/Tucumán/I1972/2012	6	2012-08-10	SIAT2/MDCK1	2560	640	2560	2560	5120	5120	5120	5120
A/Corrientes/67 120/2012	6	2012-08-15	SIAT2/MDCK1	5120	1280	2560	2560	5120	5120	5120	5120
A/Santa Fe/915 526/2012	6	2012-08-17	SIAT2/MDCK1	5120	1280	2560	2560	5120	5120	5120	5120
A/Catamarca/R4297/2012	6	2012-08-17	SIAT2/MDCK1	2560	1280	2560	2560	5120	5120	2560	5120
A/Santa Fe/1667/2012	6	2012-08-21	SIAT2/MDCK1	2560	1280	2560	2560	5120	5120	5120	5120
A/Misiones/7214/2012	6	2012-08-22	MDCK2 /MDCK1	5120	1280	2560	2560	5120	5120	5120	5120
A/Tucumán/A382/2012	6	2012-08-23	SIAT2/MDCK1	2560	640	1280	1280	2560	2560	2560	5120
A/Tucumán/A480/2012	6	2012-08-31	MDCK1/MDCK1	5120	1280	2560	2560	5120	5120	5120	5120
A/Santa Fe/1532/2012	6	not known	MDCK2/MDCK1	2560	640	1280	1280	2660	2560	2560	5120
				Vaccine 2012, 2013, 2014							

E, Egg; cell type, MDCK or SIAT; number of passages required to generate isolate/subsequent number of passages to produce sufficient virus for HA/HI analyses.

Homologous titres are indicated by underlined italics.

**Table 2.** Antigenic analyses of influenza A(H3N2) viruses (guinea pig RBC with 20 nm oseltamivir)

				Haemagglutination inhibition titre										
				Post-infection ferret antisera										
Virus		Collection date	Passage history	A/Perth16/09 F35/11	A/Victoria/208/09 F7/10	A/Alabama/5/10 F27/10	A/Stockholm/18/11 F28/11	A/Iowa/19/10 F15/11	A/Victoria/361/11 Egg F5/12	A/Berlin/93/11 F11/12	A/Victoria/361/11 T/C F14/12	A/Athens/112/12 F11/12	A/Ohio/2/12 F27/12	A/S. Australia/30/12 F28/12

	Genetic group …					5	3A	6	3C	3C	3C	3B	3B	3A
**Reference viruses**														
A/Perth/16/2009		2009-07-04	E3/E2	*1280*	80	160	160	160	160	640	640	640	160	160
A/Victoria/208/2009		2009-06-02	E3/E2	1280	*5120*	1280	2560	5120	5120	5120	5120	5120	1280	5120
A/Alabama/5/2010	5	2010-07-13	MDCK1/C2/SIAT2	<	<	*80*	80	80	40	160	320	320	40	40
A/Stockholm/18/2011	3A	2011-03-28	MDCK2/SIAT5	40	40	80	*320*	80	160	320	640	320	160	160
A/Iowa/19/2010	6	2010-12-30	E3/E2	160	320	320	640	*1280*	640	1280	1280	1280	320	320
A/Victoria/361/2011	3C	2011-10-24	E3/E2	320	640	320	160	640	*5120*	1280	640	320	160	640
A/Berlin/93/2011	3C	2011-12-07	MDCK3/SIAT4	80	80	160	320	320	320	*1280*	1280	640	320	320
A/Victoria/361/2011	3C	2011-10-24	MDCK2/SIAT2	80	80	160	320	320	320	640	*1280*	640	320	320
A/Athens/112/2012	3B	2012-02-01	SIAT5	80	160	160	320	320	320	640	1280	*1280*	320	320
A/Ohio/2/2012	3B	2012-03-08	E4/E1	160	80	640	320	640	160	640	1280	640	*1280*	160
A/South Australia/30/2012	3A	2012-05-10	E2/E1	320	160	640	640	640	320	640	1280	640	640	*320*
**Test viruses**														
A/Buenos Aires/1 004 423/20 112	3C	2012-07-24	SIAT2/SIAT1	160	160	320	640	640	640	1280	1280	1280	640	320
A/Buenos Aires/10 435 982/2012	3C	2012-07-27	SIAT2/SIAT1	160	160	320	640	320	320	1280	1280	1280	320	320
				Vaccine 2012					Vaccine 2013					

E, Egg; cell type, MDCK or SIAT (C = cell type unspecified); number of passages required to generate isolate/subsequent number of passages to produce sufficient virus for HA/HI analyses.

< = <40, the ferret reference number is given under the virus name.

Homologous titres are indicated as underlined italics.

**Table 3.** Antigenic analyses of influenza B viruses (Victoria lineage)

			Haemagglutination inhibition titre							
			SS	Post infection ferret sera						
Virus	Collection date	Passage history	B/Brisbane/60/08 SH 523	B/Malaysia/2506/04 F28/05	B/England/393/08 F5/11	B/Brisbane/60/08 F22/12	B/Paris/1762/08 F17/11	B/Hong Kong/514/09 F13/10	B/Odessa/3886/10 F19/11	B/Malta/636 714/11 F33/11
**Reference viruses**										
B/Malaysia/2506/2004	2004-12-06	E3/E5	1280	*640*	40	160	<	<	10	160
B/England/393/2008	2008-08-29	E1/E1	2560	80	*320*	640	40	40	80	640
B/Brisbane/60/2008	2008-08-04	E4/E3	*1280*	80	320	*640*	40	40	80	320
B/Paris/1762/2008	2009-02-09	C2/MDCK1	5120	10	20	80	*80*	80	0·8	20
B/Hong Kong/514/2009	2009-10-11	MDCK1/MDCK3	5120	<	20	80	80	*80*	320	20
B/Odessa/3886/2010	2010-03-19	MDCK2/MDCK1	2560	<	20	40	80	80	*160*	20
B/Malta/636 714/2011	2011-03-07	E4/E1	2560	80	320	640	40	40	80	*640*
**Test viruses**										
B/Buenos Aires/770/2012	2012-07-06	MDCK2 /MDCK1	5120	<	20	80	160	80	160	40
B/Formosa/V2367/2012	2012-08-06	MDCK1/MDCK1	5120	<	40	80	160	80	80	40
B/Tucumán/I2010/2012	2012-08-15	MDCK1/MDCK1	2560	10	40	80	80	80	80	40
B/Santa Fe/915 530/2012	2012-08-21	MDCK1/MDCK1	2560	<	20	80	80	80	80	20
B/Tucumán/A458/2012	2012-08-28	MDCK1/MDCK1	2560	<	20	80	80	80	80	20
B/Buenos Aires/R146/2012	not known	MDCK1/MDCK1	5120	10	40	80	160	160	160	40
B/Santa Fe/335 651/2012	not known	MDCK2 /MDCK1	2560	<	40	80	80	80	80	40
B/Buenos Aires/R335/2012	not known	MDCK1/MDCK1	2560	10	40	80	80	80	80	40
B/Tucumán/A423/2012	not known	Cx/MDCK2	2560	<	40	80	80	80	80	40
						Vaccine 2012, 2013*, 2014*				

SS, Hyperimmune sheep antiserum; E, Egg; cell type, MDCK or SIAT (C = cell type unspecified); number of passages required to generate isolate (x = unknown number)/subsequent number of passages to produce sufficient virus for HA/HI analyses.

< = <10, the sheep/ferret reference number is given under the virus name.

* For use in quadrivalent vaccines including both influenza B lineages.

Homologous titres are indicated as underlined italics.

**Table 4.** Antigenic analyses of influenza B viruses (Yamagata lineage)

				Haemagglutination inhibition titre										
				SS	Post-infection ferret sera									
Virus		Collection date	Passage history	B/Florida/4/06 SH479	B/Egypt/144/05 F3/07	B/Florida/4/06 F1/10	B/Brisbane/3/07 F21/12	B/England/145/08 F12/11	B/Bangladesh/3333/07 F25/08	B/Wisconsin/1/10 F7/12	B/Stockholm/12/11 Egg F34/11	B/Estonia/55 669/11 F26/11	B/Serbia/1894/11 F25/11	B/Stockholm/12/11 T/C F8/12

	Genetic group …			1	1	1	2	3	3	3	3	2	3	3
**Reference viruses**														
B/Egypt/144/2005	1	2005-05-01	E3/E5	5120	*640*	1280	1280	320	640	320	640	160	20	640
B/Florida/4/2006	1	2006-12-15	E3/E3	*5120*	320	*2560*	1280	640	640	320	640	160	20	640
B/Brisbane/3/2007	2	2007-09-03	E2/E1	5120	160	640	*1280*	160	320	160	640	160	10	320
B/England/145/2008	3	not known	Ex/E4	640	<	160	80	*320*	40	40	80	<	<	80
B/Bangladesh/3333/2007	3	2007-08-07	E4/E4	5120	80	640	320	80	*320*	80	640	<	20	640
B/Wisconsin/1/2010	3	2007-08-07	E3/E1	1280	80	320	160	160	160	*160*	160	10	20	320
B/Stockholm/12/2011	3	2007-08-07	E4/E1	5120	160	640	320	160	320	160	*640*	10	40	640
B/Estonia/55 669/2011	2	2011-03-14	MDCK2/MDCK2	2560	80	320	160	80	20	20	160	*640*	80	40
B/Serbia/1894/2011	3	2011-03-08	MDCK1/MDCK4	5120	160	320	160	320	160	80	320	160	*320*	320
B/Stockholm/12/2011	3	2011-03-28	Cx/MDCK4	5120	80	320	160	160	160	80	640	160	320	*320*
**Test viruses**														
B/Tucumán/A285/2012	2	2012-08-14	MDCK1/MDCK1	2560	40	160	80	40	10	20	160	640	20	20
B/Buenos Aires/8833/2012	2	2012-08-26	MDCK1/MDCK2	1280	<	80	40	20	10	10	80	320	20	20
										Vaccine 2013				

SS, Hyperimmune sheep antiserum; E, egg; cell type, MDCK or SIAT (C = cell type unspecified); number of passages required to generate isolate (x = unknown number)/subsequent number of passages to produce sufficient virus for HA/HI analyses.

< = <10, the sheep/ferret reference number is given under the virus name.

Homologous titres are indicated by underlined italics.

#### Influenza A(H1N1)pdm09 viruses

**Fig. 2.**
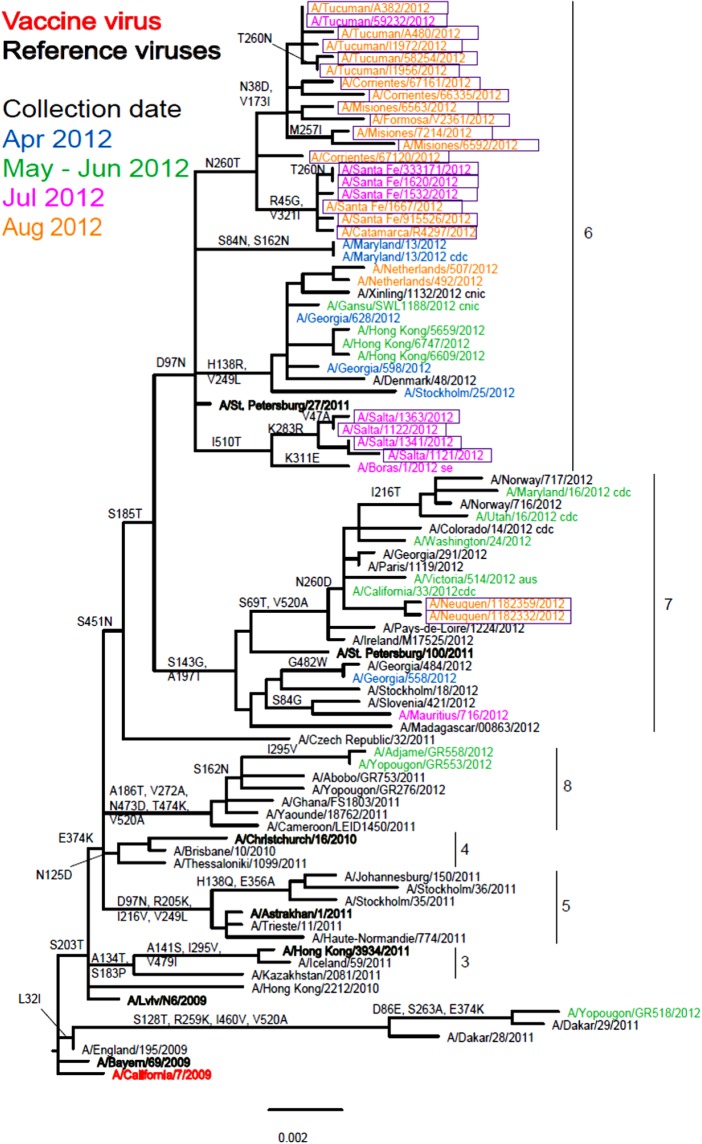
Phylogenetic comparison of influenza A(H1N1)pdm09 HA genes. Vaccine virus is indicated in bold red font and reference viruses (those to which post-infection ferret antisera have been raised) are shown in bold black font. Coloured font is used to indicate the month of clinical specimen collection for viruses isolated covering April to August 2012. Isolates from National Influenza Centre, Argentina are boxed. The majority of sequences used in the phylogenetic analysis were generated by WHO CC, London (all available in GISAID), while others were downloaded from GISAID as indicated (e.g. cnic, cdc, aus). The scale bar indicates the proportion of nucleotide changes between sequences.

Of the 26 isolates received by the WHO CC, 25 were recovered but only 20 propagated to sufficient HA titre to allow HI assays to be performed (Table 1). Nineteen of the Argentinian viruses showed a very similar reactivity pattern to each other and reacted well with the whole panel of antisera. The reactivity pattern of A/Salta/1122/2012, which carries the D156D/N polymorphism in HA1, more closely resembled those of the reference viruses A/Bayern/69/2009 and A/Lviv/N6/2009 which carry the G155E substitution and G155E/G polymorphism, respectively, in their HA1 component. Phylogenetic analysis based on 25 HA sequences showed that 23 fell in genetic group 6 with the remaining two falling in genetic group 7 (Fig. 2). The genetic group 6 viruses clearly split into two subgroups with four viruses from Salta being defined by amino acid substitutions K283R (HA1) and I510T (HA2) and the larger group being defined by N260T (HA1) substitution and showing further subdivision based on HA1 substitutions N38D with V173I, and R45G with V321I. The two genetic group 7 viruses and three of those falling in genetic group 6 could not be assayed by HI due to insufficient HA titres. Phylogenetic trees including 24 of the Argentinian virus NA sequences (data not shown) showed congruency in terms of the genetic groupings with HA phylogeny.

#### Influenza A(H3N2) viruses

**Fig. 3.**
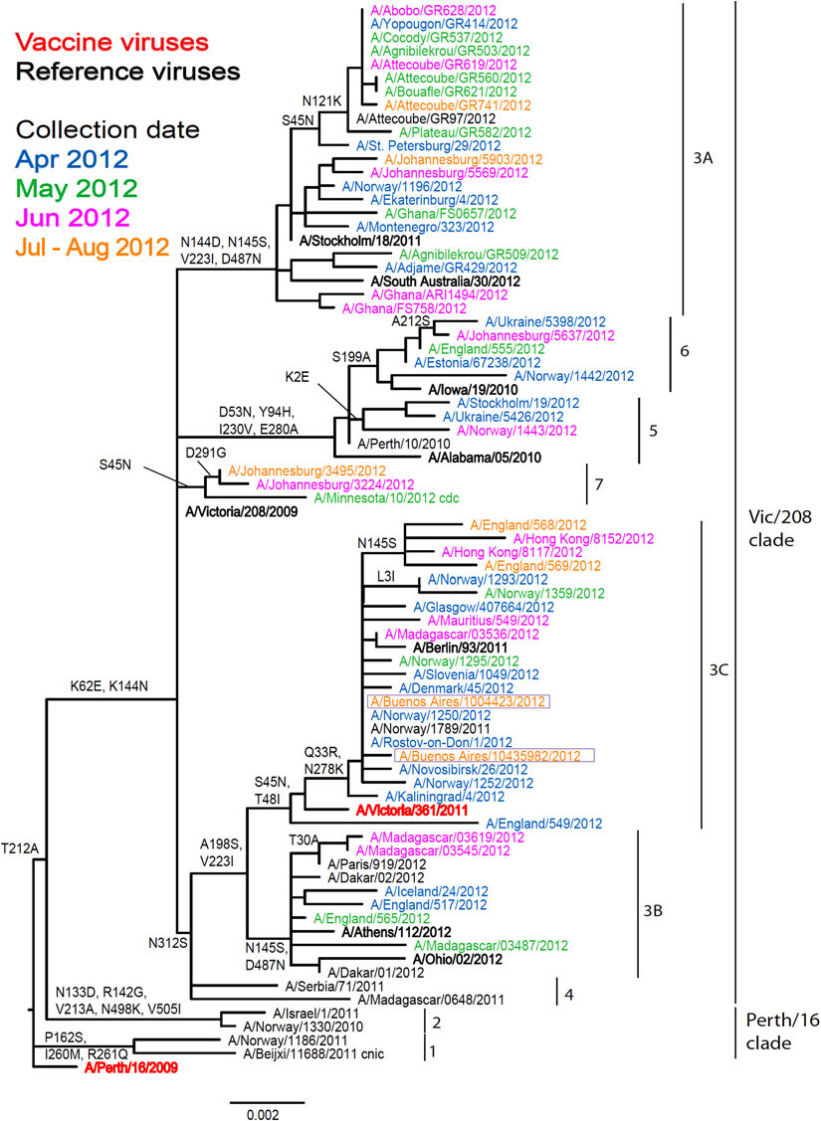
Phylogenetic comparison of influenza A(H3N2) HA genes. Vaccine virus is indicated in bold red font and reference viruses (those to which post-infection ferret antisera have been raised) are shown in bold black font. Coloured font is used to indicate the month of clinical specimen collection for viruses isolated covering April to August 2012. Isolates from National Influenza Centre, Argentina are boxed. The majority of sequences used in the phylogenetic analysis were generated by WHO CC, London (all available in GISAID), while others were downloaded from GISAID as indicated (e.g. cnic, cdc, aus). The scale bar indicates the proportion of nucleotide changes between sequences.

The two Argentinian A(H3N2) viruses showed poor reactivity with post-infection ferret antisera raised against three egg-propagated viruses in terms of reduction, i.e. eightfold or greater reduction in HI titre compared to the titres with the homologous viruses: the 2012 A/Perth/16/2009, and 2013 A/Victoria/361/2011 vaccine viruses and A/Victoria/208/2009 (Table 2). However, in terms of absolute titre, reactivity was somewhat better with antiserum raised against A/Victoria/361/2011. The two test viruses reacted better with antisera raised against the egg-propagated A/Iowa/19/2010, A/Ohio/2/2012 and A/South Australia/30/2012 reference viruses compared to the homologous titres. These antisera, particularly those raised against A/South Australia/20/2012, had lower homologous titres than other recent egg-propagated H3N2 viruses. Both test viruses showed good reactivity with post-infection ferret antisera raised against the five tissue-culture propagated reference viruses, which included A/Victoria/361/2011. Phylogenetic analyses of HA (Fig. 3) and NA genes (data not shown) demonstrated that the two test viruses fell into the A/Victoria/208/09 clade, genetic group 3C for both genes.

#### Influenza B viruses

**Fig. 4.**
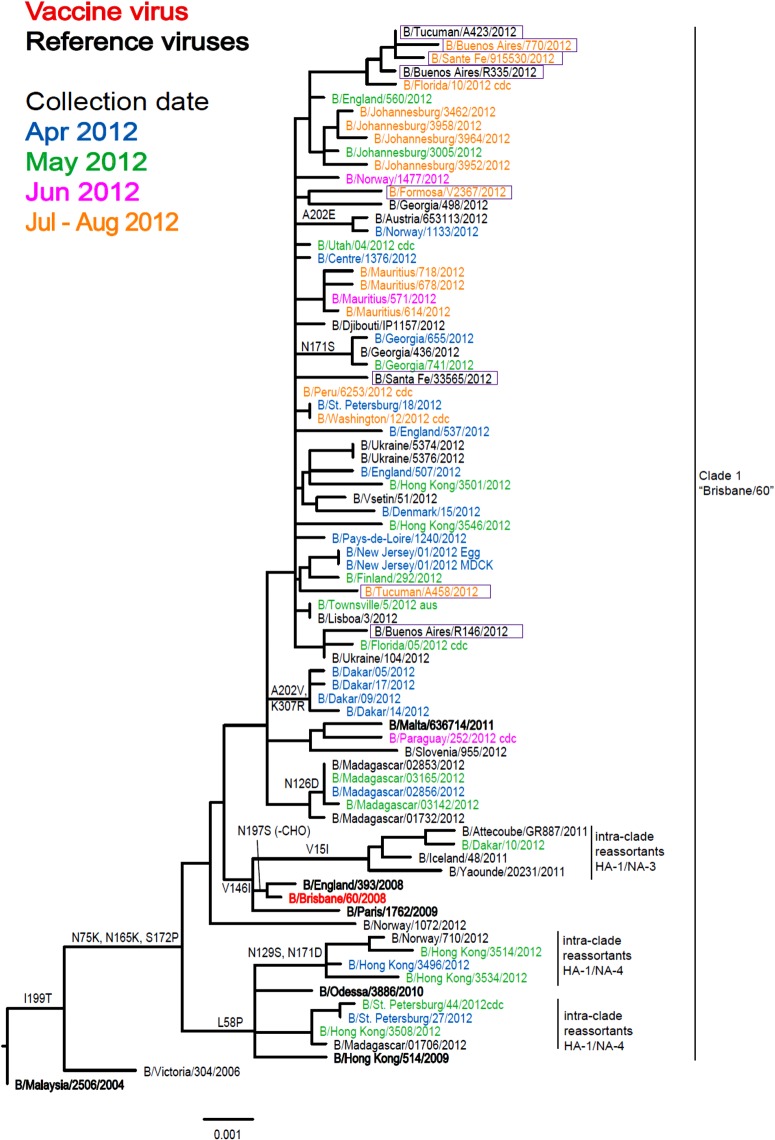
Phylogenetic comparison of influenza B (Victoria lineage) HA genes. Vaccine virus is indicated in bold red font and reference viruses (those to which post-infection ferret antisera have been raised) are shown in bold black font. Coloured font is used to indicate the month of clinical specimen collection for viruses isolated covering April to August 2012. Isolates from National Influenza Centre, Argentina are boxed. The majority of sequences used in the phylogenetic analysis were generated by WHO CC, London (all available in GISAID), while others were downloaded from GISAID as indicated (e.g. cnic, cdc, aus). The scale bar indicates the proportion of nucleotide changes between sequences.

**Fig. 5.**
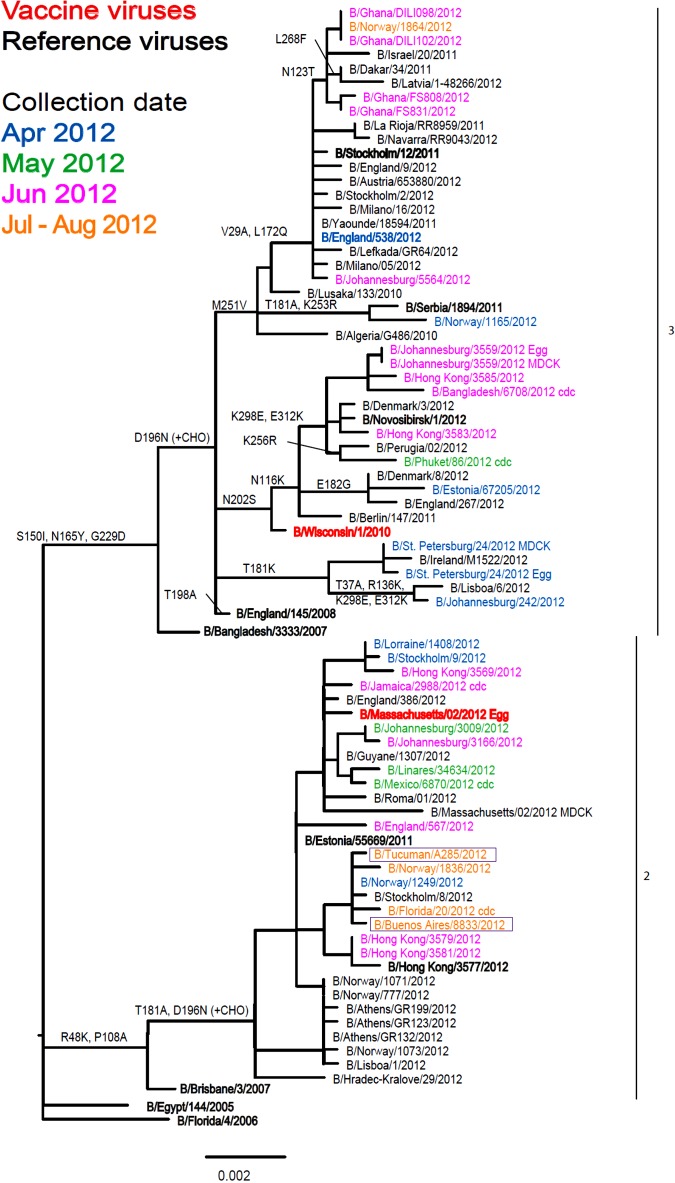
Phylogenetic comparison of influenza B (Yamagata lineage) HA genes. Vaccine virus is indicated in bold red font and reference viruses (those to which post-infection ferret antisera have been raised) are shown in bold black font. Coloured font is used to indicate the month of clinical specimen collection for viruses isolated covering April to August 2012. Isolates from National Influenza Centre, Argentina are boxed. The majority of sequences used in the phylogenetic analysis were generated by WHO CC, London (all available in GISAID), while others were downloaded from GISAID as indicated (e.g. cnic, cdc, aus). The scale bar indicates the proportion of nucleotide changes between sequences. For three virus sequences derived from solely egg or MDCK, cell isolated and propagated viruses are included.

Of the 11 influenza B viruses studied, nine belonged to the Victoria lineage (Table 3 and Fig. 4) and two to the Yamagata lineage (Table 4 and Fig. 5).

All tested viruses belonging to the Victoria lineage gave the common pattern of reactivity that has been observed over the course of the previous two seasons: the viruses reacted with an eightfold reduced titre with antiserum raised against egg-propagated vaccine virus, B/Brisbane/60/2008, compared to the homologous titre (Table 3). However, viruses reacted at titres similar to the homologous viruses with antisera raised against influenza B viruses genetically closely related to the vaccine virus but propagated in cells (B/Paris/1762/2008, B/Hong Kong/514/2009 and B/Odessa/3886/2010). Low reactivity was also seen with antiserum raised against the egg-propagated virus B/Malta/636 714/2011 compared to the homologous titre. Phylogenetic analyses including eight HA and seven NA (data not shown) genes from the Argentinian viruses showed that all belonged to the B/Brisbane/60/2008 genetic clade (Clade 1), with four of the eight/seven clustering together (Fig. 4).

The two Yamagata lineage viruses reacted well with antiserum raised against B/Estonia/55 669/2011 and poorly with antiserum raised against the virus recommended for the 2013 vaccine, B/Wisconsin/1/2010 (Table 4). Generally both test viruses showed poor reactivity, eightfold or greater reduction in HI titre, with all antisera raised against the other eight reference viruses compared to the respective homologous titres, the one exception being B/Tucuman/A285/2012 which showed a fourfold drop with antiserum raised against egg-propagated B/Stockholm/12/2011. Phylogenetic analyses of HA and NA (data not shown) genes demonstrated that both test viruses belonged to clade 2, represented by the reference virus B/Estonia/55 669/2011.

### Antiviral susceptibility results

Phenotypic testing to assess virus susceptibility to the NAIs, oseltamivir and zanamivir, was performed on a number of test viruses: 16 A(H1N1)pdm09, two A(H3N2) and 10 influenza B. The A(H3N2) and influenza B viruses showed normal inhibition by both antivirals. Of the 16 A(H1N1)pdm09 viruses studied, 15 showed normal inhibition by both antivirals but one, A/Salta/1341/2012, showed highly reduced inhibition by oseltamivir (IC_50_: 187·9 nm) and reduced inhibition by zanamivir (IC_50_: 8·3 nm). NA gene sequencing of this isolate and the original clinical specimen showed both carried the amino acid substitution N295S, known to reduce the inhibitory effects of the NAIs studied [22]. The clinical specimen was taken from a 17-year-old pregnant outpatient with symptom onset on 25 July 2012 who had not been vaccinated; on 26 July 2012 oseltamivir treatment was initiated.

## DISCUSSION

Clinical surveillance, supported by antigenic and genetic analyses are invaluable in guiding effective influenza control measures. The influenza pandemic caused by A(H1N1)pdm09 underscored the importance of surveillance networks that can rapidly characterize circulating viruses for effective response and containment [23].

The present study reports results of virologi**c**al and drug susceptibility surveillance in selected influenza A(H1N1)pdm09, A(H3N2), and B viruses isolated in Argentina in 2012 when types A and B influenza viruses of both subtypes and lineages co-circulated in the human population throughout the study period.

The transmission pattern of influenza viruses was different in the four countries of the Southern Cone in South America in 2012. Increased viral circulation in the area was first noted at the end of April to early May in Chile and Paraguay; Argentina and Uruguay begun to report active transmission in early June. Surprisingly, peak activities were detected very late in this region. In Argentina the peak was registered at the beginning of August, 10 weeks later than in the last 9 years [24].

In Argentina, before emergence of A(H1N1)pdm09, seasonal influenza vaccination was recommended and funded for individuals aged from 6 months having chronic co-morbidities, pregnant woman and the elderly population aged ⩾65 years [25, 26]. Following the analysis of the pandemic, the Argentinean MoH considered it necessary to include among the risk groups all children aged <5 years [27]; since 2011 this recommendation has been changed to include children aged from 6 months to 2 years only.

All influenza A(H1N1) viruses detected globally since January 2010 have been related to A(H1N1)pdm09. Despite considerable genetic variation in circulating A(H1N1)pdm09 viruses, very little antigenic drift has been observed since the 2009 pandemic, and the majority of the Argentinean isolates analysed in this report were closely related to A/California/7/2009, the recommended vaccine virus since 2009. The low reactivity observed in A/Salta/1122/2012 was associated with a polymorphism at amino acid position 156 of HA1, the normal aspartic acid being present as a mixture of aspartic acid and asparagine. Changes in the 153–157 region of HA1 have been identified in many cell-propagated A(H1N1)pdm09 viruses, notably those isolated in SIAT cells, showing low reactivity with post-infection ferret antisera raised against A/California/7/2009, that are not present in the corresponding clinical specimens [10].

The two A(H3N2) test viruses showed clear antigenic divergence from the 2012 Southern Hemisphere vaccine virus, A/Perth/16/2009, based on HI assay. They showed significantly better reactivity with antisera raised against five cell-propagated reference viruses, notably those four with collection dates in 2011 and 2012 which included the cell-propagated prototype of A/Victoria/361/2011. An A/Victoria/361/2011-like virus was recommended for the Southern Hemisphere 2013 influenza season.

As observed globally, in Argentina both lineages of influenza type B co-circulated with the Victoria lineage being prevalent. The B/Victoria lineage test viruses were antigenically and genetically closely related to cell-propagated surrogates of the 2012 vaccine virus, B/Brisbane/60/2008-like virus, as assessed by HI assay.

A virus of the influenza B Yamagata lineage was not included in the 2012 trivalent vaccine but this lineage, although increasing in number in respect of both viruses in genetic clades 2 and 3, remained the minority lineage in 2012. Given the continued rise in the proportion of B Yamagata lineage viruses, WHO recommended a clade 3 B/Wisconsin/1/2010-like Yamagata virus for use in the 2013 season [24]. The two clade 2 Yamagata lineage viruses analysed here showed poor reactivity with antiserum raised against B/Wisconsin/1/2010 and a range of other clade 3 viruses. Both viruses reacted well with antiserum raised against cell-propagated B/Estonia/55 669/2011, a clade 2 virus (Table 4). The continued rise in the proportion of Yamagata lineage viruses worldwide, notably for clade 2, led to an amended recommendation for the 2014 season of a clade 2 B/Massachusetts/2/2012-like virus [28].

Globally, a limited number of sporadic oseltamivir-resistant cases of A(H1N1)pdm09 have been reported in the early phases of the pandemic, [29] and most remain sensitive to NAIs. Subtype-specific NA mutations in framework or catalytic residues that confer resistance to the antiviral drugs have been reported *in vitro* and *in vivo* [30, 31]. NA-H275Y is the most frequent change related to resistance to oseltamivir in A(H1N1) and A(H5N1) [32, 33]. The first cases of oseltamivir-resistance in A(H1N1)pdm09 viruses were reported for specimens collected in Denmark and Japan. The NA-N295S mutation was originally reported in drug-resistant H5N1 viruses [34]. In the course of the present study we detected an A(H1N1)pdm09 virus, A/Salta/1341/2012 showing highly reduced inhibition by oseltamivir and reduced inhibition by zanamivir which carried an NA-N295S substitution. In terms of influenza antiviral susceptibility surveillance detection of N1 NA-H275Y is screened for routinely. A disadvantage of following this methodology is the possibility of failing to detect other less frequent changes that could affect the susceptibility to antiviral drugs. While there are a number of instances of resistant viruses being isolated from persons who had either been treated or been in close contact with an individual who had been treated with oseltamivir, this virus was detected in an untreated hospitalized patient. Determining the origins and genesis of such drug-resistant viruses, notably in the absence of drug pressure, the understanding of the emergence and persistence of oseltamivir resistance in relation to the evolution of influenza viruses, drug use and pandemic preparedness, is important. The NIC, together with the MoH, gives guidelines for the notification, vaccination, diagnosis, management and specific treatment of the acute respiratory infections at the beginning of each year, since the pandemic A(H1N1)pdm09. For the specific influenza treatment, the MoH acquires and distributes NAIs and provides guidelines for their administration. This situation emphasizes the need for ongoing virological surveillance to determine the susceptibility of local strains and detect emerging resistance to these antivirals.

Surveillance of antiviral resistance is an essential tool in the clinical management of severe or complicated influenza cases and is an important firstline defence during a probable pandemic [35].

The Argentina NIC together with other Southern Hemisphere NICs has contributed to the WHO's decision to review, twice a year, the composition of the influenza vaccine.

This report highlights the importance of NICs performing antigenic and genetic surveillance of influenza viruses in order to update the national ministries of health recommendations and contributes to the WHO twice yearly update on the composition of influenza vaccines. Additionally, data obtained by NICs contributes to global influenza surveillance, which serves as the primary global alert mechanism for detecting the emergence of novel influenza viruses that could cause public health alerts and result in pandemics. Overall, such studies allow countries to update measures for prevention and control of influenza as emerging situations dictate.
